# Diurnal and photoperiodic changes in thyrotrophin‐stimulating hormone β expression and associated regulation of deiodinase enzymes (*DIO2, DIO3*) in the female juvenile chicken hypothalamus

**DOI:** 10.1111/jne.12554

**Published:** 2017-12-18

**Authors:** I. C. Dunn, P. W. Wilson, Y. Shi, D. W. Burt, A. S. I. Loudon, P. J. Sharp

**Affiliations:** ^1^ Roslin Institute and Royal (Dick) School of Veterinary Studies Roslin Scotland UK; ^2^ College of Animal Science and Veterinary Medicine Henan Agricultural University Zhengzhou China; ^3^ UQ Genomics Initiative University of Queensland Saint Lucia Qld Australia; ^4^ Faculty of Life Sciences University of Manchester Manchester UK

**Keywords:** pars tuberalis, photoperiodism, reproduction, thyroid

## Abstract

Increased thyrotrophin‐stimulating hormone β (*TSH*β) expression in the pars tuberalis is assumed to be an early step in the neuroendocrine mechanism transducing photoperiodic information. The present study aimed to determine the relationship between long‐photoperiod (LP) and diurnal *TSH*β gene expression in the juvenile chicken by comparing LP‐photostimulated birds with groups kept on a short photoperiod (SP) for 1 or 12 days. *TSH*β expression increased by 3‐ and 23‐fold after 1 and 12 days of LP‐photostimulation both during the day and at night. Under both SP and LP conditions, *TSH*β expression was between 3‐ and 14‐fold higher at night than in the day, suggesting that *TSH*β expression cycles in a diurnal pattern irrespective of photoperiod. The ratio of *DIO2/3* was decreased on LPs, consequent to changes in *DIO3* expression, although there was no evidence of any diurnal effect on *DIO2* or *DIO3* expression. Plasma prolactin concentrations revealed both an effect of LPs and time‐of‐day. Thus, *TSH*β expression changes in a dynamic fashion both diurnally and in response to photoperiod.

## 1 INTRODUCTION

Increased thyrotrophin‐stimulating hormone β (*TSH*β) expression in the pars tuberalis is assumed to be an early step in the neuroendocrine mechanism transducing photoperiodic information to the reproductive axis, driving thyroid hormone conversion in adjacent ependymal tanycyte cells of the ventral hypothalamus.^1^ In mammals, photoperiodic information is considered to be interpreted via the nocturnal melatonin signal, as secreted by the pineal acting via the melatonin 1 receptor (MT1) on *TSH*β expressing cells,^2^ and this sets the phase of transcription factor Eya3 expression.^3^ In avian species, despite a functional pineal system with several biological effects,^4^, ^5^ the photoperiodic information for reproduction is assumed to be translated by deep brain photoreceptors,^6^, ^7^ although there is evidence indicating that the importance of melatonin may depend on the output under investigation.^8^ In these cases, a circadian clock mechanism for measuring daylength situated in the medial basal hypothalamus has been demonstrated.^9^ In quail and sheep, EYA3 expression is induced on long days and, at least in mammals, is inhibited by melatonin. EYA3 is a transcriptional activator of the *TSH*β promoter and fulfills the requirements to be part of the internal coincidence model proposed to explain the interaction of light and the circadian system.^10^ The pars tuberalis is known to regulate other seasonal hormones, such as prolactin via paracrine regulation of adjacent lactotroph cells, with this not involving thyroid hormone conversion. In mammals, a number of pars tuberalis‐specific signals have been proposed as prolactin regulators^11^ (S. Wood and A. S. I. Loudon, unpublished data), whereas, in birds, an alternative mechanism involving changes in clock genes *Per2* and *Cry1* in the pars tuberalis acting on vasoactive intestinal polypeptide neurones has been suggested.^12^ The initial discovery of the response of *TSH*β to photostimulation in the Japanese quail 1 was preceded by observations of what we now consider to be downstream effectors of this system, including the demonstration that exogenous thyroxine (T4) activates reproduction^13^ consequent upon photostimulated conversion of T4 to triodothyronine.^14^ Observations in a closely‐related *Galliform,* the chicken, have been sparse, although T4 injection results in the stimulation of gonadotrophin‐releasing hormone (GnRH) expression and gonadotrophin release.^15^ An increase in *TSH*β expression has been reported to occur during the first long day and *DIO2* after 2 weeks of long‐photoperiod (LP) exposure.^16^

If *TSH*β expression is driven by an internal circadian clock mechanism, we might expect to see differences in the expression of *TSH*β at different times of the day. We therefore assessed the diurnal expression of *TSH*β under a short photoperiod (SP) and following long‐term photostimulation, as well as downstream genes *DIO2* and *DIO3*, in domesticated chickens. Earlier studies have defined photo‐inductive effects of LP on gonadotrophin secretion, with a critical daylength of around 11 hours^17^ and an increase in *GNRH1* expression post‐photostimulation.^18^ Chickens also exhibit an acute day 1 response to LP of luteinising hormone (LH) release,^15^ similar to that observed in quail. In the present study, we investigated whether the dynamic photoperiodic inductive effects observed in the quail are replicated in domesticated chickens and also whether the differences in response of these 2 closely‐related species might underlie domestication events or their evolution.

## 2 MATERIALS AND METHODS

### 2.1 Animals and sampling

#### 2.1.1 Experiment 1

Female 1‐day‐old Isa Brown chicks, a commercial hybrid layer chicken (*Gallus gallus domesticus)* were maintained for 2 days on LP (14:10 hour light/dark cycle) prior to transfer to a climate controlled chamber on SP (8:16 hour light/dark cycle). They remained there until 5 weeks of age when they were randomised between 2 chambers at the same time as remaining on a SP (8:16 hour light/dark cycle). At 7 weeks of age, the photoperiod for 1 chamber was changed to LP (16:8 hour light/dark cycle) by extending dusk. The next day, hens (n = 7) were killed 4 hours after lights on from both chambers. Additional hens (n = 7) were killed 4 hours after lights off from both chambers. After 12 days, further hens (n = 7) were killed 4 hours after lights on from both chambers and more hens (n = 7) were killed 4 hours after lights off from both chambers. The age and sex of the birds used was based on previous experiments where the most robust responses to photostimulation in prepubertal hens had been observed^17^, ^18^, ^19^, ^20^

Prior to killing by an overdose of barbiturate, a blood sample was taken from the brachial vein and the birds were weighed. Immediately after death, the birds were dissected to remove the pituitary and the basal hypothalamus including the pars tuberalis and median eminence. The mean ± SD mass of the basal hypothalamus dissection was 4.1 ± 0.1 mg and the pituitary mass is reported in the results. These tissues were immediately frozen in liquid nitrogen before being stored at −80°C prior to RNA extraction. The ovary and oviduct were also removed and weighed.

#### 2.1.2 Experiment 2

In Experiment 1, we observed no change in *DIO2* expression related to photoperiod, unlike in the quail.^1^ To confirm the ability of our DIO2 assay to detect dynamic changes in expression, an experiment was performed using quail bred at Roslin Institute derived from a population originally kept by Professor Brian Follett.^21^ The protocol was similar in terms of lighting to Experiment 1, except only 12 days of photostimulation was used and dissections were performed only 4 hours after lights off (n = 8). The quail were photostimulated at 3 weeks of age to match their earlier sexual maturity.

Experiments were carried out under the Animals (Scientific Procedures) Act 1986*,* project license 70/7909 and individual experiments were approved by the institutional ethics committee.

### 2.2 Measurement of gene expression

Total RNA was extracted from the tissue samples using Ultraspec II reagent (AMS Biotechnology, Abingdon, UK) and Lysing Matrix D tubes in a FastPrep Instrument (MP Biomedicals, Cambridge, UK). Total RNA treated with DNAse was reverse transcribed using a *Not*I‐(dT)18 primer and a First‐Strand cDNA synthesis kit (GE Healthcare, Little Chalfont, UK) in accordance with the manufacturer's instructions. cDNA samples were diluted x15 for use in a real‐time polymerase chain reaction (PCR).

Quantitative PCR for estimation of levels of gene expression were made using specific primers (Table 1) designed using Primer3^22^ to amplify products of approximately 100‐200 bp in length crossing intron/exon boundaries in the *TSH*β, *DIO2* and *DIO3* genes. In the case of the primers for *TSH*β, *DIO2* and *DIO3*, the primers were designed to work on chicken cDNA and quail cDNA. Note that *DIO3* is intronless. For primers, the chromosomal position and sequence are provided in Table 1. PCR products from the amplification of cDNA with the gene specific primers were purified for standards using a QIAEX II gel extraction kit (Qiagen Ltd, Crawley, UK) and their concentration was measured using a NanoDrop spectrophotometer (Thermo Fisher Scientific, Loughborough, UK). Serial dilutions of standards were made to create standard curves for real‐time PCR quantification. Real‐time PCR reactions were run on an MX3000p real‐time PCR machine (Agilent Technologies, Cheadle, UK) under the conditions: 95°C for 2 minutes, 40 cycles of 95°C for 15 seconds and 60°C for 30 seconds. Real‐time PCR reactions (25 μL) were run using 10 μL of cDNA template together with SYBR green master mix (VHBio Ltd, Gateshead, UK) and gene specific primers (100 nmol L^‐1^). Samples and standard curves were run in duplicate on the same 96‐well plate along with water blank controls. Standards were diluted to produce top standards detectable after approximately 15 PCR cycles. Assays were analysed using mxpro (Agilent Technologies) and expression was normalised using a weighted average of lamin B receptor (*LBR*), β‐actin and glyceraldehyde‐3‐phosphate dehydrogenase expression. In the case of the quail experiment, only *LBR* was used as a control gene. There was no evidence of any treatment effects for the control gene expression in an ANOVA (for primers, chromosomal position and sequence, see Table 1).

**Table 1:** Primers used in the quantification of mRNA by a quantitative reverse transcriptase‐polymerase chain reaction

Primer	Genome position galGal4 build	Primer sequence
pitTSHF051	Chromosome 26: 3 922 240‐3 922 260	CTCTTTGGCCTGACTTTTGG
pitTSHR242	Chromosome 26: 3 923 984‐3 924 004	TGTGCACACGTTTTGAGACA
DIO2CQF2	Chromosome 5: 39 837 655‐39 837 670	CGCCTACAAGCAGGTCAAAC
DIO2CQR1	Chromosome 5: 39 826 745‐39 826 769	CACACTTGCCACCAACACTCTT
DIO3F3	Chromosome 5: 49 200 868‐49 200 888	AGGCTCTCTTCCTTCGGGAT
DIO3R3	Chromosome 5: 49 200 947‐49 200 967	TAGCACTTGCTAGGCAGCAC
GAPDHpw_F2	Chromosome 1: 76 435 973‐76 435 993	ACGGTGGATGGCCCCTCTGG
GAPDHpw_R2	Chromosome 1: 76 436 550‐76 436 570	GGCCCATCAGCAGCAGCCTT
LBR_F	Chromosome 3: 16 759 889‐16 759 911	GGTGTGGGTTCCATTTGTCTACA
LBR_R	Chromosome 3: 16 759 949‐16 759 968	CTGCAACCGGCCAAGAAA
ACT1	Chromosome 14: 4 169 819‐4 169 834	AATCAAGATCATTGCCCCAC
ACT2	Chromosome 14: 4 169 531‐4 169 550	TAAGACTGCTGCTGACACC

### 2.3 Hormone assays

Plasma prolactin and LH were measured in 1 assay using homologous radioimmunoassays as described previously.^23^, ^24^ The intra‐assay coefficient of variance was 8.4% and 10.2%, respectively.

### 2.4 Statistical analysis

The results were analysed by ANOVA in genstat, version 13 (VSN International, Hemel Hempstead, UK) using log‐transformed data to approximate to a normal distribution where appropriate. The significance of differences between means was calculated by least significant differences where indicated as appropriate from the ANOVA.

## 3 RESULTS

### 3.1 Body mass and reproductive organs are increased by photostimulation

Body mass or organ mass did not show any diurnal variation. Therefore, for these traits, only photostimulation (LP vs SP) and the duration of treatment (1 vs 12 days) were used as variables for ANOVA. As predicted, body mass and organ mass increased after 12 days (*F*_1,55_ = 26.92‐158.26, *P* < .001) because animals were still in the juvenile growth period. There was an effect of photostimulation on body mass (*F*_1,55_ = 7.29, *P* = .009) and, after 12 days of exposure, LP animals were heavier than those on SP (858 g vs 772 g, *P* < .05) (Table 2). Pituitary and ovary mass were significantly higher in LP animals (*F*_1,53_ = 11.65, *P* = .001 and *F*_1,54_ = 7.62, *P* = .008, respectively, when body mass was fitted as a covariate). Specifically, at 1 and 12 days, pituitary mass was greater in photostimulated birds (*P* < .01 and *P* < .001, respectively) (Table 2), as was also the case for the ovary (*P* < .01 and *P* < .05, respectively) (Table 2). In the case of the oviduct mass, the ANOVA was also significant for photostimulation (*F*_1,55_ = 12.23, *P* < .001) but oviduct mass was increased significantly (*P* < .01) only after 12 days of exposure to LP when the difference between the treatment means was tested (Table 2).

**Table 2:** Body, pituitary, ovary and oviduct mass of 7‐week‐old hens photostimulated for 1 or 12 days

Duration	1 day		12 days		ANOVA		
Photoperiod	SP	LP	SP	LP	Duration	Day‐length	Inter‐action
Body mass	602 ± 11	607 ± 18	772 ± 18	858 ± 19*	<0.001	0.009	0.018
Pituitary mass (mg)	4.5 ± 0.2	5.1 ± 0.2	5.3 ± 0.2	6.4 ± 0.2	<0.001	<0.001	0.219
Pituitary mass (mg)^a^	5.0	5.6**	5.0	5.8***	0.428	0.001	0.669
Ovary mass (g)	0.20 ± 0.01	0.25 ± 0.01	0.27 ± 0.01	0.34 ± 0.02	<0.001	<0.001	0.386
Ovary mass (g)^a^	0.26	0.30**	0.23	0.26*	0.280	0.002	0.624
Oviduct mass (g)^b^	0.13 ± 0.01	0.15 ± 0.01	0.17 ± 0.01	0.23 ± 0.03	<0.001	<0.001	0.112
Oviduct mass (g)^c^	0.15	0.17	0.15	0.18**	0.824	0.015	0.568

Data are the observed mean ± SEM and estimated values from analysis using body mass as a covariate. Significance of differences between the means for SP‐LP contrasts; *P < .05, **P < .01, ***P < .001.

a Estimated mean from ANOVA using body mass as a covariate.

b Observed values but ANOVA performed on log‐transformed values.

c Back‐transformed estimated means from ANOVA using body mass as a covariate.

### 3.2 Plasma LH varies diurnally

**Figure 1:**
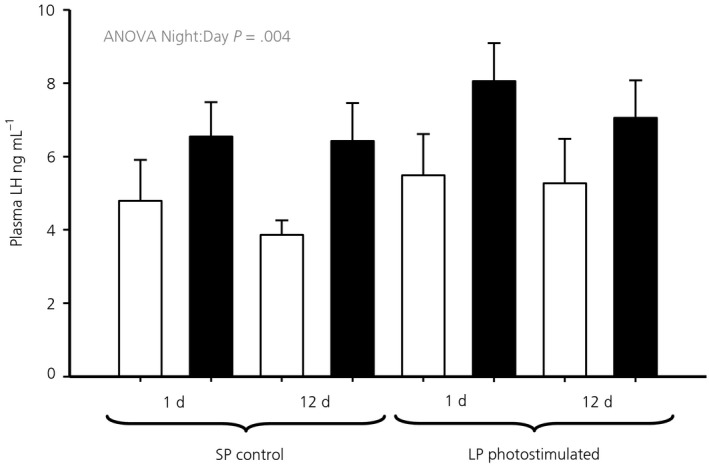
Plasma luteinising hormone concentrations in samples taken prior to death. Hens were exposed to either 1 or 12 days of a long photoperiod (LP) (16:8 hour light/dark cycle) or maintained on a short photoperiod (SP) (8:16 hour light/dark cycle). Open bars represent the samples taken during the light period (4 hours after lights on), whereas the solid bars represent the night samples (4 hours after lights off)

Plasma LH concentrations were not significantly elevated after photostimulation; however, there was a clear diurnal effect on LH concentrations (ANOVA, *F*_1,55_ = 9.13, *P* = .004) across the entire experiment (Figure 1).

### 3.3 Plasma prolactin concentrations increase after 1 day of photostimulation and vary diurnally after photostimulation

**Figure 2:**
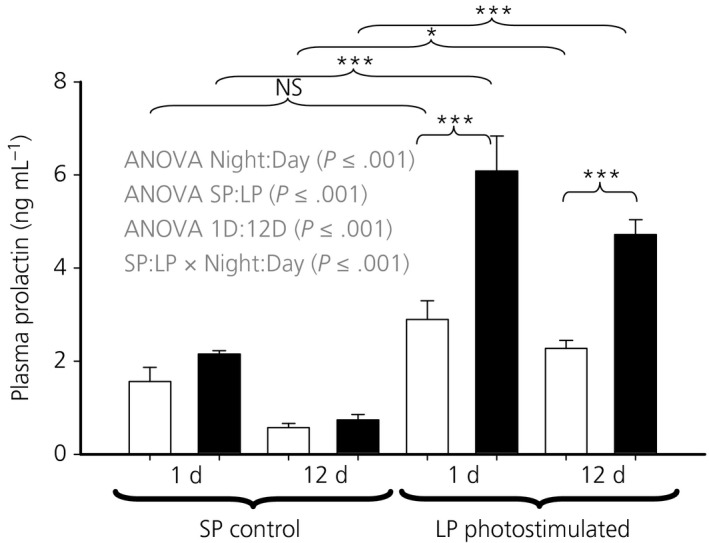
Plasma prolactin concentrations in samples taken prior to death. Hens were exposed to either 1 or 12 days of a long photoperiod (LP) (16:8 hour light/dark cycle) or maintained on a short photoperiod (SP) (8:16 hour light/dark cycle). Open bars represent the samples taken during the light period (4 hours after lights on), whereas the solid bars represent the night samples (4 hours after lights off). Specific comparisons between means. NS, not significant; *P < .05; ***P < .001

By contrast to plasma LH, plasma concentrations of prolactin were increased approximately 3‐fold after 1 day of photostimulation when comparing samples taken at night. A diurnal variation in the concentrations of prolactin was evident, as indicated by the interaction of photostimulation and time of sample (ANOVA, *F*_1,55_ = 212.56, *P* < .001), with an approximately 2‐fold difference between day and night samples (Figure 2).

### 3.4 Diurnal variation in *TSHβ* expression occurs regardless of whether photostimulated or not

**Figure 3:**
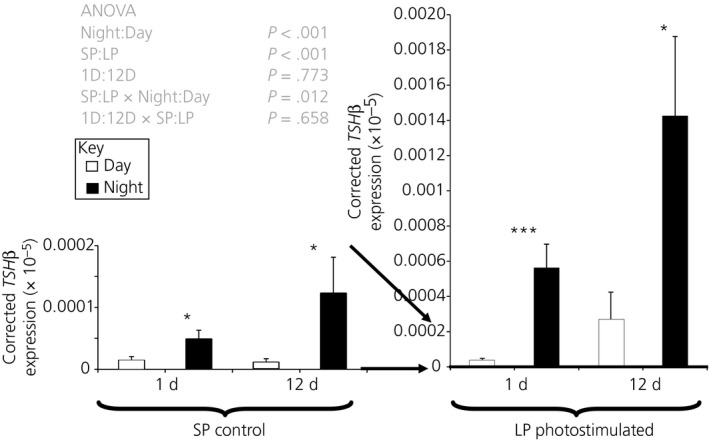
Thyrotrophin‐stimulating hormone β (*TSH*β) expression in the basal hypothalamus. Hens were exposed to either 1 or 12 days of a LP (16L:8D) or maintained on SP (8L:18D) Open bars represent the samples taken during the light period (4 hours after lights on), whereas the solid bars represent the night samples (4 hours after lights off). Specific comparisons between means are indicated for night‐day comparisons *P < .05; ***P < .001. All other contrasts are significant at least at *P* < .05. Note that the 2 panels are on different scales as indicated by the arrows

*TSH*β expression varied diurnally (ANOVA, *F*_1,55_ = 29.76, *P* < .001) regardless of whether photostimulated or not (Figure 3) and the differences between the individual means were all significant (*P* < .05). The night/day fold change in *TSH*β expression was 3 and 11 at 1 and 12 days, respectively, in the SD control and 14 and 5 at 1 and 12 days, respectively, in the LD photostimulated groups. Photostimulation increased both the day and night values for *TSH*β expression (ANOVA, *F*_1,55_ = 69.91, *P* < .001) and there was an interaction between whether the sample was taken during the day or night and with photostimulation (ANOVA, *F*_1,55_ = 6.88, *P* = .012) (Figure 3). The magnitude of the differences between LP and SP groups after 12 days was a 23‐fold increase in expression during the day (*P* < .001), which is greater than the 11‐fold difference observed during the night (*P* < .01).

### 3.5 *DIO3* but not *DIO2* expression responds to photostimulation but shows no diurnal variation

**Figure 4:**
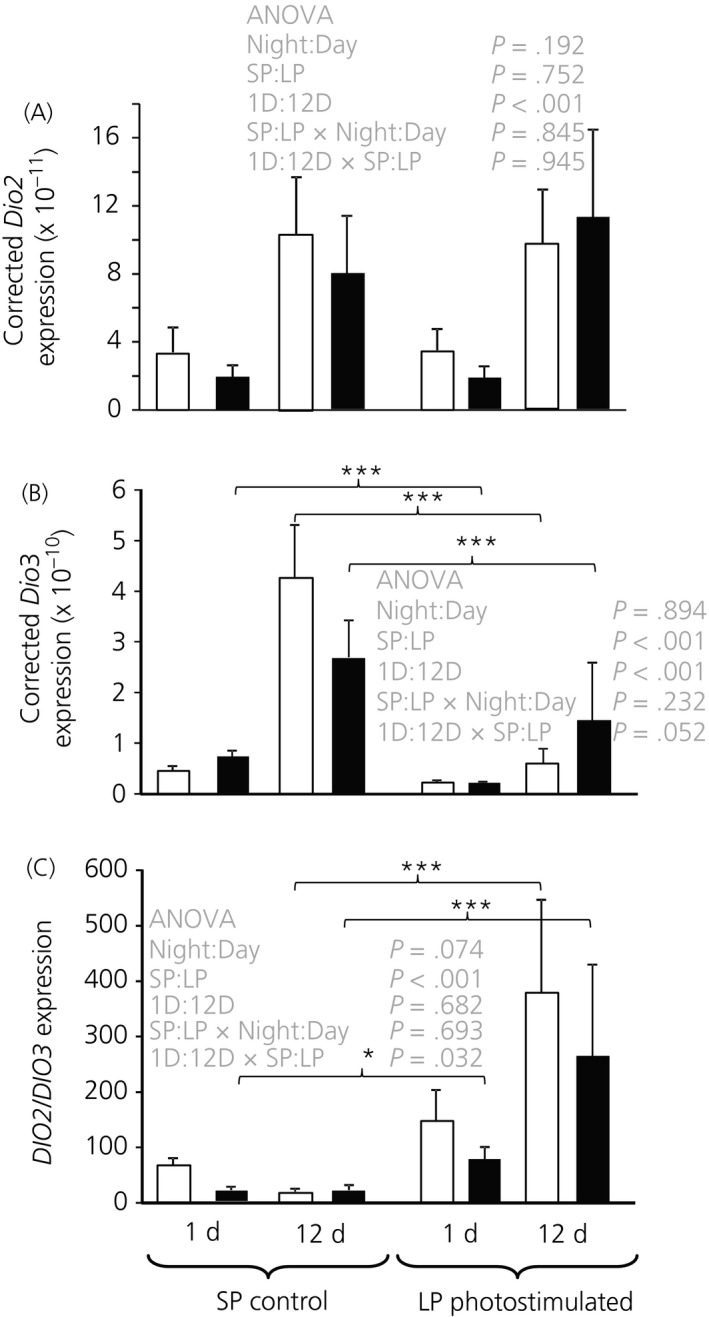
(A), *DIO2*, (B), *DIO3* expression and (C) their ratio in basal hypothalamus. Hens were exposed to either either 1 or 12 days of a long photoperiod (LP) (16:8 hour light/dark cycle) or maintained on a short photoperiod (SP) (8:16 hour light/dark cycle). Open bars represent the samples taken during the light period (4 hours after lights on), whereas the solid bars represent the night samples (4 hours after lights off). Specific comparisons between means are indicated for SP‐LP comparisons, *P < .05; ***P < .001. There were no significant night day effects

Measurement of *DIO2* did not reveal any discernible pattern of expression attributable to time of day or photostimulation (Figure 4A) but did increase with age (ANOVA, *F*_1,55_ = 16.92, *P* < .001). The samples taken after 12 days of photostimulation had approximately 3.7 times the level of expression observed at 1 day, which corresponds to approximately 7 and 9 weeks of age.

By contrast, *DIO3* expression (Figure 4B) was clearly reduced after photostimulation (ANOVA, *F*_1,55_ = 37.76, *P* < .001) and *DIO3* was expressed at a higher level in the 12‐day exposure group than in the 1‐day group (ANOVA, *F*_1,55_ = 27.64, *P* < .001) as was the case for *DIO2*. There was also a tendency for the photostimulation effect to be stronger after 12 days of exposure to LP than after 1 day, as indicated by the day × photostimulation interaction (ANOVA, *F*_1,55_ = 3.96, *P* = .052). A significant effect of day length was observed after only 1 day of exposure to LP in the night samples (*P* < .001), although the difference was not significant in the day samples.

The ratio of DIO2 to DIO3 is likely to be important in driving photoperiodic responses (Figure 4C). This emphasised the significant effect of photoperiod (ANOVA, *F*_1,55_ = 33.37, *P* < .001) and interaction between photostimulation and the duration of exposure or age of the bird (ANOVA, *F*_1,55_ = 4.86, *P* = .032) on the balance of expression of these genes. The photoperiodic effect was most evident following 12 days of exposure. There was no evidence of a diurnal effect on the *DIO2*/*DIO3* expression ratio.

### 3.6 *DIO2* expression is increased in quail after photostimulation

**Figure 5:**
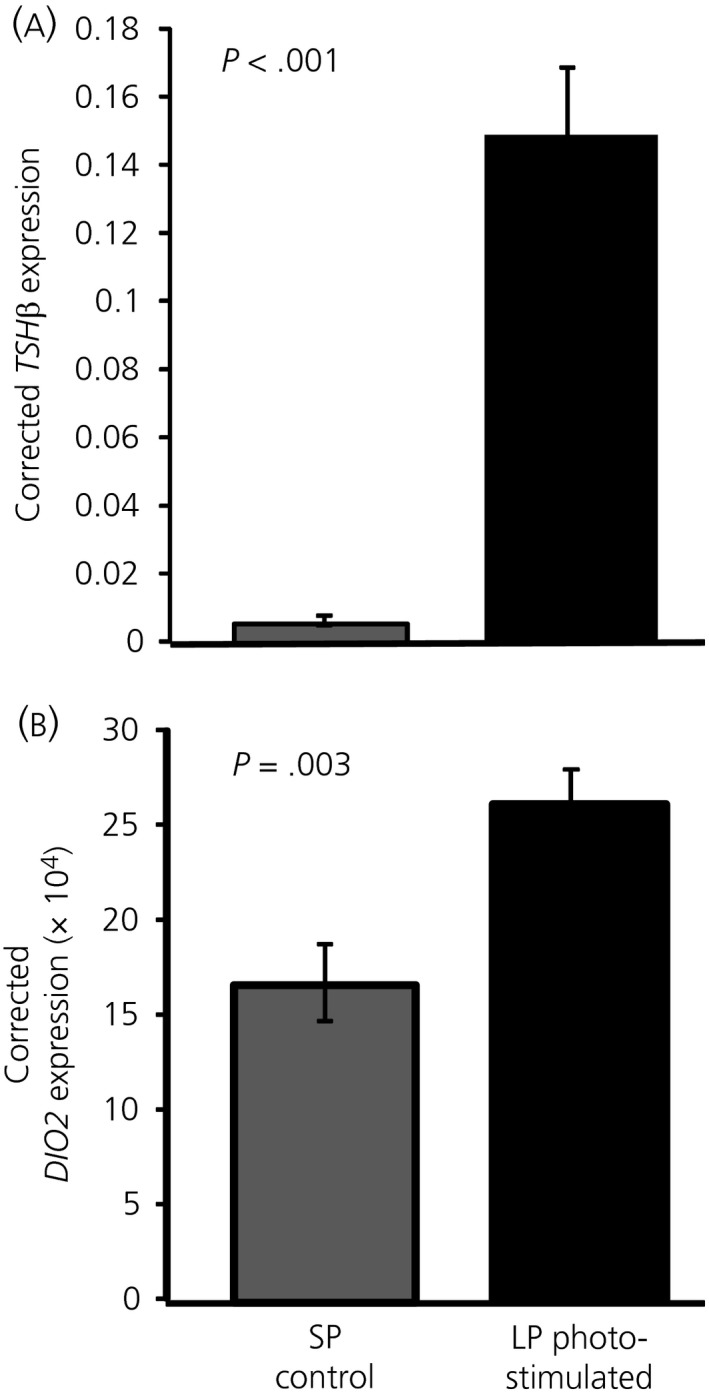
Thyrotrophin‐stimulating hormone β (*TSH*β) and *DIO2* expression in quail basal hypothalamus. Quail were exposed to either a long photoperiod (LP) (16:8 hour light/dark cycle) or maintained on a short photoperiod (SP) (8:16 hour light/dark cycle). Dissections were performed 4 hours after lights off

To confirm the lack of effect observed in *DIO2* expression in the chicken, we aimed to confirm that our assay can detect differences in the quail hypothalamus after photostimulation. The *DIO2* primers were selected to work on both species and an increase in expression in the quail after 12 days of photostimulation was observed, as well as an increase in *TSH*β expression (Figure 5).

## 4 DISCUSSION

We have demonstrated that juvenile domesticated chickens have as robust a photoperiodic response in terms of *TSH*β expression and changes in the ratio of *DIO2/3* expression as that previously observed in quail.^1^ However, in the present study, there is no evidence that DIO2 is affected by photostimulation in juvenile chickens as it is in quail. We have observed considerably higher *TSH*β expression in LPs compared to the respective SP birds. Equally striking was that differences in *TSH*β expression between day and night samples on both LP and SPs. There is a 3‐ and 11‐fold difference in *TSH*β expression between the night and day sample in 1 day and 12 day birds on SP, respectively. This was proportionally as large as the fold difference produced by photostimulation. Thus, *TSH*β expression in the pars tuberalis cycles daily, regardless of whether the bird was photostimulated or not.

In domesticated chickens, the region of the genome containing *TSHR* and *DIO2* and *DIO3* has been under intense selection 25, 26 and a genetic association at the locus was observed in a study on incubation behaviour and persistency.^27^ Therefore, there may be a link between the locus, photoperiod and reproduction that may have increased fecundity during domestication. Although there is evidence for differences in *TSH*β expression with selection, it is only in the context of declining photoperiod,^28^ and so, currently, we can only speculate on the involvement of these genes and domestication, although it does provide a testable hypothesis. The fact that the response of *DIO2* expression does not appear to respond to photoperiod in juvenile chickens but does change with age might be a suitable place to start. This is especially the case given that hens bred for egg production, although influenced by light, do start reproductive activity on short days.^29^

Studies describing the link between *TSH*β in the pars tuberalis and the photoperiodic response in quail,^1^ revealed that expression of *TSH*β was increased 14 hours after lights on, returning to baseline 6 hours later, although only in photostimulated conditions. There was no evidence of an increase in *TSH*β prior to or during the nocturnal phase on SPs.^1^ In the mouse, a species not classically considered as photoperiodic, the results clearly show the levels of *TSH*β mRNA and protein are higher during the dark phase than the light phase^30^ but with an amplitude less than that observed in the experiment reported in the present study. This was abolished in mice lacking the MT1 receptor.^30^ Further studies on the MT1 null mice demonstrated the signalling through the receptors drove expression of *PER1, CRY1, CLOCK* and *BMAL1*, suggesting the involvement of circadian signalling in the pars tuberalis.^31^ Similarly, in rats, *TSH*β gene expression changes diurnally, again with modest amplitude compared to that observed in the chicken in these experiments. Administration of melatonin to pinealectomised rats caused suppressed *TSH*β expression during both the day and night, with pinealectomy alone suppressing *TSH*β expression during the dark period.^32^

If *TSH*β is a read out of night and day then nocturnal increases in melatonin, might provide the signal for this. However, counter intuitively, in these experiments, *TSH*β is elevated on LP, when the night is shorter, yet *TSH*β is higher at night. A clue to explaining the paradox may lie in rodents where melatonin inhibited *TSH*β expression but the effect takes time to develop.^32^ If melatonin has a slow onset inhibitory effect on *TSH*β then shorter nights would increase base line *TSH*β expression on LP. This complements recent observations in a migratory bird (*Emberiza melanocephala*) where *TSH*β expression on LP is highest before lights off and declines through the dark period, although there was no evidence for such a rhythm on short days.^33^ This was taken as evidence that photoperiodically‐induced alterations in clock gene rhythms may be linked to the observed changes in *TSH*β. The results reported in the present on the chicken support this observation, although the existence of the phenomenon on short days suggests the possibility that an internal circadian clock mechanism may drive *TSH*β expression irrespective of photoperiod in chickens. In any case, there is a complex interaction between clock genes, their phase and measures of the length of the dark period such as melatonin on the expression of *TSH*β that underlies diurnal, photoperiodic and, ultimately, seasonal events,^10^ which we are likely to be observing another facet of in the present study. The debate on the relative roles of circadian pacemakers and melatonin in driving seasonal processes in birds has suggested a balance of effects of the 2 mechanisms depending on the system studied 8 and it may be that the factors driving the expression of the genes investigated in the present study also feature components of both systems.

The absence of an increase in LH after photostimulation in the experiments reported in the present study was not unexpected. The demonstration of a photoperiodic response curve at this age required pre‐handling and 6 samples per hen.^17^ However, a significant diurnal effect was evident for LH over the whole experiment. The prolactin response to photostimulation is more robust 34 and, in the present study, we observed a persistent and significantly higher level at night after a single long day on LP. Prolactin concentrations were significantly different between night and day after 1 or 12 days on LP. Both a diurnal pattern in circulating LH and prolactin have previously been observed in chickens.^35^, ^36^

We could speculate that, in birds, prolactin might be ultimately controlled by the *TSH*β system in the pars tuberalis because of the correlation between their release and expression and, similarly, the diurnal variation of LH. The major problem with this hypothesis is the belief that photoperiod induced LH secretion is driven through GnRH release by changes in the *DIO2/3* enzyme ratio. Although we observed a change in the ratio of *DIO2/3* expression in the night sample after a single long day, there is no evidence for a diurnal effect on the *DIO2/3* expression ratio. Of course, this may be because the fluctuating *TSH*β signal is integrated into a linear *DIO2/3* response. Whatever the link between *TSHβ* and reproduction we should not think of *TSH*β expression as a static entity but, instead, as a dynamic system that changes diurnally and in response to photoperiod.

In conclusion, we have confirmed changes in the expression of *TSH*β and the ratio of *DIO2*/*3* in response to increased photoperiod in juvenile domestic chickens and we have also shown that *TSH*β but not *DIO2/3* shows a very large diurnal variation of similar magnitude to that of the response to photostimulation.

## CONFLICT OF INTERESTS

The authors declare that they have no conflicts of interest.
